# Machine learning–based risk stratification for gastrointestinal bleeding in ICU patients with cirrhosis: evidence from the MIMIC database

**DOI:** 10.3389/fmed.2025.1701973

**Published:** 2025-12-05

**Authors:** Yuxin Duan, Weifan Sui, Zefeng Cai, YimaoXua Xia, Jianyun Li, Jianhua Fu

**Affiliations:** Department of Interventional Radiology, The Affiliated People’s Hospital of Jiangsu University, Zhenjiang, Jiangsu, China

**Keywords:** cirrhosis, gastrointestinal bleeding, machine learning, in-ICU, prediction model

## Abstract

**Background:**

In critically ill patients with cirrhosis, gastrointestinal bleeding (GIB) is a common complication that significantly impacts clinical outcomes during ICU hospitalization. Early identification of high-risk patients is crucial for preventing complications and guiding appropriate clinical interventions, which can improve treatment outcomes.

**Objective:**

To develop and externally validate a machine learning model for predicting in-hospital GIB in ICU patients with cirrhosis, identify key predictors, and assess its clinical utility for risk stratification and decision-making.

**Methods:**

A retrospective cohort study was conducted, including 3,160 ICU patients diagnosed with cirrhosis from the Medical Information Mart for Intensive Care IV (MIMIC-IV) database. Patients were divided chronologically into training (*n* = 2,528) and testing (*n* = 632) cohorts based on their ICU admission dates. External validation was performed on a separate cohort of 523 ICU patients with cirrhosis extracted from the publicly available Electronic Intensive Care Unit (EICU) database. Key predictive variables were identified through a combination of the Boruta algorithm, correlation analysis, and variance inflation factor (VIF) assessment, ensuring both predictive relevance and control of multicollinearity. Six ML algorithms—logistic regression, k-nearest neighbors, support vector machine, random forest (RF), multilayer perceptron, extreme gradient boosting, and gradient boosting machine—were trained and evaluated through 10-fold cross-validation. Model performance was rigorously assessed based on the area under the receiver operating characteristic curve (AUC-ROC), accuracy, sensitivity, specificity, F1-score, calibration curves, and decision curve analysis (DCA). Shapley additive explanations (SHAP) analysis was employed to interpret and rank variable importance. Additionally, multivariable logistic regression models were constructed to elucidate the relationship between anticoagulant therapy and the incidence of in-ICU GIB after comprehensive adjustment for relevant clinical factors.

**Results:**

Among the ML algorithms evaluated, the RF model achieved AUC of 0.86 (95% CI: 0.84–0.88) in the training cohort and 0.72 (95% CI: 0.68–0.76) in the test cohort, with sensitivity 0.68, specificity 0.71, and precision 0.47. The key predictors identified by the model included red blood cell count, hemoglobin level, platelet count, and anticoagulant therapy, all of which were significantly associated with the risk of gastrointestinal bleeding. Decision curve analysis indicated that the RF model provides meaningful clinical utility for early risk stratification. Multivariable logistic regression further revealed that anticoagulant use independently correlated with a lower risk of in-ICU GIB (or: 0.29; 95% confidence interval: 0.24–0.34). Stratified analyses based on gender, age, weight, and additional subgroups consistently confirmed the robustness of the protective association between anticoagulant therapy and reduced GIB risk.

**Conclusion:**

The RF model demonstrated stable discrimination for predicting GIB risk in ICU patients with cirrhosis across multiple cohorts. Built on readily available clinical data, it enables timely risk stratification and informs individualized preventive interventions in critical care settings.

## Introduction

Liver cirrhosis is a common outcome of chronic liver disease and remains a major contributor to global mortality and morbidity. Despite changes in its underlying causes over the past decade, cirrhosis continues to result in serious complications such as hepatic encephalopathy, ascites, variceal bleeding, and hepatocellular carcinoma, and was responsible for approximately 2.4% of all global deaths (1). Furthermore, a substantial proportion of hospitalized patients with liver cirrhosis require ICU admission, which imposes considerable economic burdens on both families and healthcare system (2, 3). GIB, a frequent complication in patients with liver cirrhosis, significantly worsens clinical outcomes and remains a major determinant of poor prognosis.

Existing studies have demonstrated a strong association between liver cirrhosis and the occurrence of GIB, particularly among patients with portal hypertension and compromised liver function (4–7). Among hospitalized patients with liver cirrhosis, GIB presents a major clinical challenge, often leading to treatment delays and substantially elevated in-hospital mortality. However, the underlying mechanisms of GIB in patients with liver cirrhosis remain incompletely understood. Proposed contributors include portal hypertension–related variceal rupture, anticoagulation-related mucosal injury, coagulopathy, and systemic inflammatory responses (8). Especially in patients with liver cirrhosis requiring anticoagulant and antiplatelet treatment, maintaining an optimal balance between thrombosis prevention and bleeding risk remains particularly challenging, thereby further increasing the likelihood of GIB.

Previous studies have primarily focused on identifying risk factors for upper gastrointestinal bleeding (UGIB) in patients with cirrhosis, such as ascites and spleen stiffness and other liver function–related parameters. However, some of these studies were limited by small sample sizes, lack of external validation, and insufficient consideration of time-dependent variables such as the initiation and duration of anticoagulant or antiplatelet therapy (9–11). ML approaches may improve risk prediction by capturing non-linear relationships not detected by traditional regression models.

This study combined machine learning approaches with traditional statistical methods to identify risk factors for GIB in cirrhotic patients after ICU admission. The primary goals were to develop and externally validate a robust predictive model and to evaluate the impact of GIB on short-term outcomes, with the goal of enabling earlier intervention in high-risk patients.

## Materials and methods

This retrospective cohort study utilized data from the MIMIC-IV version 3.1 database, a large publicly available critical care dataset. The EICU database was employed for external validation to assess the generalizability of the predictive model. As a large, single-center, publicly available critical care database, the MIMIC database contains comprehensive and high-quality data on patients admitted to the intensive care units (ICUs) of Beth Israel Deaconess Medical Center (Boston, MA, United States) between 2008 and 2019 (12). This study was approved by the Institutional Review Boards of both Beth Israel Deaconess Medical Center and the Massachusetts Institute of Technology (Cambridge, MA, United States). Access to the MIMIC databases is granted to individuals who have completed the Collaborative Institutional Training Initiative (CITI) program. One author (Yuxin Duan) completed the required course and were granted access (certification number: 68219045). The study was reported in accordance with the REporting of studies Conducted using Observational Routinely Collected Health Data (RECORD) statement.

Patients diagnosed with liver cirrhosis were identified based on the International Classification of Diseases, 9th and 10th revisions (ICD-9/10). The diagnosis of gastrointestinal bleeding (GIB) was based on the following ICD codes: ICD-9 codes 4560, 45620, 53021, 5307, 53082, 53100, 53140, 53240, 53783, 53784, 56202, 56212, 56213, 5693, 56985, 5780, 5781, 5789, and ICD-10 codes I8501, I8511, K250, K252, K254, K256, K280, K284, K286, K922, K921. Exclusion criteria were as follows: (1) age < 18 years at the first ICU admission; (2) multiple ICU admissions, with data from only the first admission retained for analysis; (3) GIB before ICU admission; (4) presence of malignancy; and (4) ICU length of stay < 24 h. Ultimately, 3,160 patients were included in the final analysis, with an additional 523 patients from the EICU database used for external validation.

The software PostgreSQL (version 16.9) and Navicat Premium (version 16) were used to extract information with a running Structured Query Language (SQL). The extraction of potential variables could be divided into four main groups: (1) demographics, such as age, gender, weight. (2) Comorbidities, including hypertension (HTN), chronic obstructive pulmonary disease (COPD) acute kidney injury (AKI), sepsis, chronic kidney disease (CKD), ischemic heart disease (IHD). (3) Laboratory indicators, including red blood cells (RBC), white blood cells (WBC), hemoglobin (HGB), platelet (PLT), red cell distribution width (RDW), serum potassium, serum sodium, serum creatinine (Cre), blood urea nitrogen (BUN), alanine aminotransferase (Alt), Aspartate aminotransferase (Ast), total bilirubin (TBIL), international normalized ratio (INR), prothrombin time (pt). (4) Severity of illness scores at admission, including the Acute Physiology Score III (APSIII), the simplified Acute Physiology Score II (SAPS-II), Oxford Acute Severity of Illness Score (OASIS), the Sepsis-related Organ Failure Assessment score (SOFA), the Glasgow Coma Scale (GCS), and Systemic Inflammatory Response Syndrome (SIRS), Child-Turcotte-Pugh score (CTP), Model for End-Stage Liver Disease (MELD, MELD = 3.78 × ln(TBIL) + 11.2 × ln(INR) + 9.57 × ln(Cre) + 6.43), and Model for End-Stage Liver Disease– Sodium (MELD-Na, MELD-Na = MELD + 1.32 × (137−Na) −[0.033 × MELD × (137−Na)]) scores were calculated for each patient based on their individual laboratory and clinical parameters. (5) Medication use following ICU admission, including antibiotics, glucocorticoids, diuretics, anticoagulants, and antiplatelet agents. All predictor variables, including laboratory values, disease severity scores, and medication exposures, were extracted from data generated within the first 24 h after ICU admission.

To avoid potential bias, variables with more than 20% missing data were excluded from the analysis. For variables with less than 20% missingness, multiple imputation was performed using the predictive mean matching (PMM) method implemented via the “mice” package in R (Supplementary Figure 1 and Supplementary Table 1) (13, 14).

### Model construction and validation

In this study, feature selection from the training dataset was performed using the Boruta algorithm, a robust wrapper method built upon random forest (RF) classifiers. This technique evaluates the relevance of each variable by comparing its importance score (z-score) against that of artificially created “shadow” features (15). These shadow features are generated by duplicating the original variables and randomly permuting their values. A variable is retained as important if its z-score consistently exceeds the highest z-score among the shadow features across multiple iterations. Features identified as important through this process were subsequently used for model development. Features selected by Boruta were further assessed for multicollinearity using correlation analysis and VIF. Variables with strong correlations (*r* > 0.8) or high VIF values (> 10) were reviewed and adjusted to ensure model robustness and interpretability (16).

Key variables identified by the Boruta algorithm were incorporated into six distinct ML algorithms for model development, including logistic regression (LR), multilayer perceptron (MLP), support vector machine (SVM), RF, extreme gradient boosting (XGBoost), and gradient boosting machine (GBM) (17). To ensure optimal performance across all ML models, hyperparameter optimization was performed via grid search in combination with 10-fold cross-validation. A systematic evaluation of critical parameters—such as the number of estimators, the maximum depth of trees, and the minimum number of samples required at a leaf node—was conducted during the tuning process. To address class imbalance and prevent data leakage, the Synthetic Minority Over-sampling Technique combined with Edited Nearest Neighbors (SMOTEENN) was implemented within a pipeline structure, ensuring that resampling was applied only to the training folds during cross-validation (18). The procedure was iteratively performed across all cross-validation folds to determine the hyperparameter set that achieved the optimal AUC.

Model performance was primarily evaluated based on the ROC curve, with the model exhibiting the highest AUC selected for further analysis. Discriminative ability was additionally assessed using sensitivity, specificity, accuracy, and the F1-score. Model calibration was evaluated using calibration curves to compare predicted probabilities with observed outcomes. To assess potential clinical applicability, DCA was subsequently performed. The top-performing model was then subjected to interpretability analysis. After being trained on the training cohort, the model parameters were fixed and its performance was subsequently validated on both the testing and external validation cohorts.

To enhance interpretability of the top-performing model, SHAP were employed. Rooted in cooperative game theory, SHAP assigns each input feature a contribution value—known as the Shapley value—by fairly attributing the model’s output to individual predictors. This method not only quantifies feature importance, but also visualizes the direction and magnitude of each variable’s influence on the prediction, thereby offering comprehensive insight into the model’s decision logic. In this study, SHAP analysis played a key role in identifying potential risk factors for GIB in patients with cirrhosis and in validating the robustness and clinical relevance of these predictors across the testing and external validation cohorts.

Continuous variables were summarized as medians with interquartile ranges (IQR), and categorical variables were presented as frequencies and percentages. Differences between groups were assessed using chi-square (χ^2^) tests or Fisher’s exact tests for categorical variables, and Wilcoxon rank-sum tests for continuous variables. Logistic regression analyses and subgroup analyses were conducted to explore the association between anticoagulant use and the risk of GIB among patients with cirrhosis. All statistical analyses were performed using R software (version 4.3.2; R Foundation for Statistical Computing) and Python (version 3.12.7). Statistical significance was set at a two-tailed *p*-value of < 0.05.

## Results

### Participant inclusion and baseline characteristics

**FIGURE 1:**
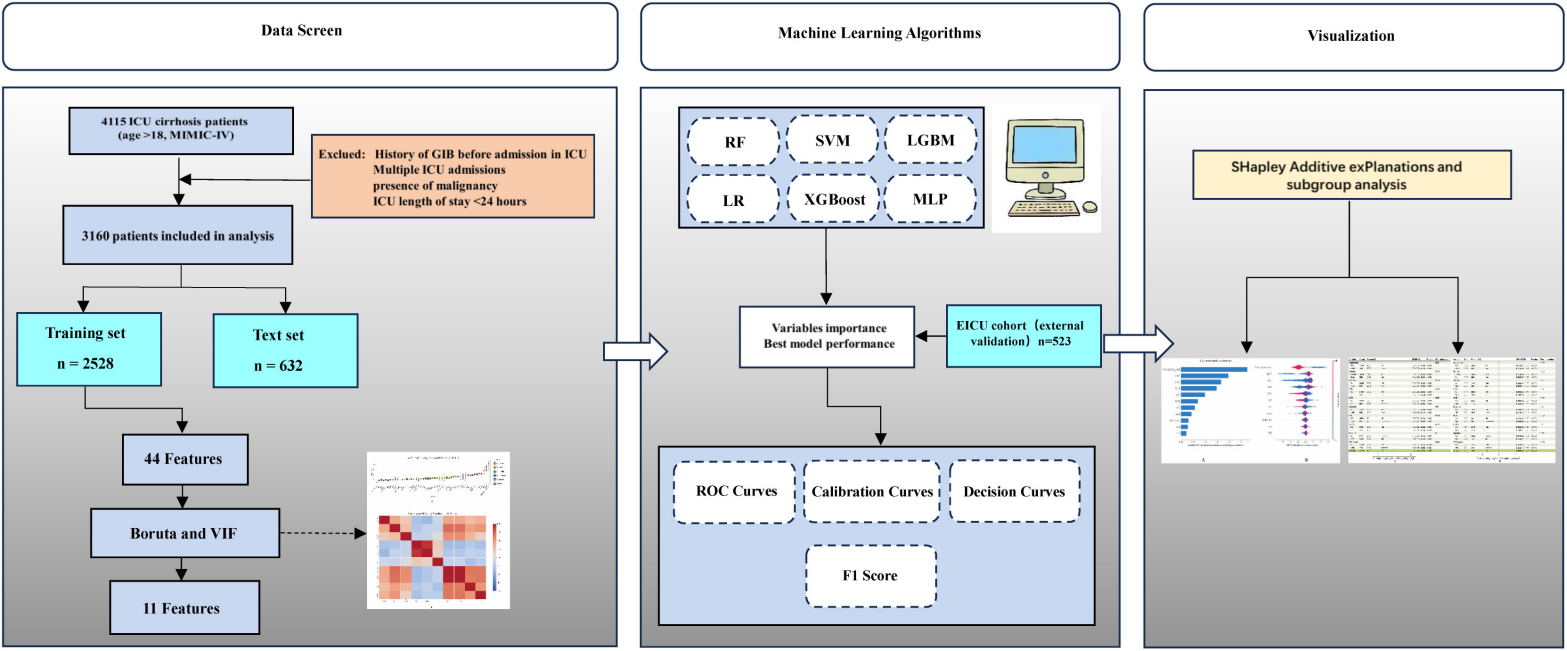
The overall flowchart of the study.

A total of 4,115 patients with cirrhosis aged over 18 years admitted to the ICU were initially identified from the MIMIC-IV database. Among these, 337 patients were excluded due to malignant tumors, and 618 were excluded because of a short ICU stay (< 24 h). Ultimately, 3,160 patients were included in the final analysis (Figure 1). Of these patients, 2,061 (65.2%) were male. During their ICU stay, 555 patients (27%) experienced GIB, while 1,506 (78%) did not. Significant differences were observed between the GIB and non-GIB groups in terms of age, vital signs (e.g., Nbpd, HR), and laboratory parameters (e.g., RDW, TBIL). Additionally, a higher proportion of patients in the GIB group met the Sepsis-3 criteria compared to the non-GIB group (71.6% vs. 28.4%). However, there was no significant difference in gender distribution between the two groups. Additional patient characteristics are detailed in Table 1. Baseline characteristics of the training and test cohorts are detailed in Supplementary Tables 2, 3, respectively.

**TABLE 1:** Baseline characteristics of patients with cirrhosis in ICU.

Variable	Overall	NO GIB	GIB	*p*-value
	*N* = 3,160	*N* = 2,311	*N* = 849	
Age, years, median (IQR)	60.00 (15.00)	60.00 (15.00)	58.00 (15.00)	< 0.001
Weight, Kg, median (IQR)	82.50 (29.30)	82.70 (30.45)	82.30 (27.20)	0.574
Gender, male, n (p%)	2,061.00 (65.22%)	1,506.00 (65.17%)	555.00 (65.37%)	0.915
**Laboratory tests, median (IQR)**				
HGB, (g/dL)	9.40 (2.70)	9.60 (2.80)	8.80 (2.60)	< 0.001
PLT, (× 10^9^/L)	102.00 (92.00)	107.00 (96.00)	92.00 (83.00)	< 0.001
RDW, (%)	16.70 (3.70)	16.40 (3.80)	17.50 (3.60)	< 0.001
RBC, (× 10^12^/L)	3.00 (0.97)	3.07 (0.98)	2.79 (0.86)	< 0.001
WBC, (× 10^9^/L)	9.50 (8.50)	9.50 (8.40)	9.50 (8.30)	0.153
Albumin, (g/dL)	3.00 (0.90)	3.00 (0.90)	2.90 (0.80)	< 0.001
K,(mmol/L)	4.20 (1.00)	4.10 (1.00)	4.20 (1.10)	< 0.001
Na, (mmol/L)	137.00 (7.00)	137.00 (7.00)	137.00 (8.00)	0.098
Alt, (U/L)	33.00 (53.00)	33.00 (61.00)	33.00 (39.00)	< 0.001
Ast, (U/L)	68.00 (116.00)	67.00 (119.00)	74.00 (108.00)	0.592
TBIL, (mg/dL)	2.60 (5.50)	2.40 (5.00)	3.60 (7.80)	< 0.001
Cre, (mg/dL)	1.20 (1.30)	1.20 (1.30)	1.20 (1.30)	0.479
BUN, (mg/dL)	25.00 (30.00)	24.00 (28.00)	30.00 (33.00)	< 0.001
INR	1.70 (0.70)	1.60 (0.80)	1.80 (0.80)	< 0.001
Pt, (s)	18.30 (8.00)	17.90 (7.90)	19.20 (8.30)	< 0.001
**Vital signs, median (IQR)**				
HR	91.00 (27.00)	90.00 (26.00)	93.00 (28.00)	0.075
Nbps, (mmHg)	116.00 (33.00)	117.00 (35.00)	115.00 (31.00)	0.030
Nbpd, (mmHg)	66.00 (22.50)	66.00 (23.00)	64.00 (22.00)	0.072
Ascites, n (p%)	825.00 (26.11%)	558.00 (24.15%)	267.00 (31.45%)	< 0.001
HE, n (p%)	299.00 (9.46%)	178.00 (7.70%)	121.00 (14.25%)	< 0.001
**Comorbidities, n (p%)**				
HTN	965.00 (30.54%)	708.00 (30.64%)	257.00 (30.27%)	0.843
AKI	1,818.00 (57.53%)	1,303.00 (56.38%)	515.00 (60.66%)	0.031
CKD	554.00 (17.53%)	419.00 (18.13%)	135.00 (15.90%)	0.144
Diabetes	938.00 (29.68%)	713.00 (30.85%)	225.00 (26.50%)	0.018
IHD	631.00 (19.97%)	487.00 (21.07%)	144.00 (16.96%)	0.010
COPD	396.00 (12.53%)	309.00 (13.37%)	87.00 (10.25%)	0.019
Sepsis3	2,342.00 (74.11%)	1,654.00 (71.57%)	688.00 (81.04%)	< 0.001
**Risk scores, median (IQR)**				
Sofa	8.00 (6.00)	8.00 (6.00)	9.00 (6.00)	< 0.001
Apsiii	54.50 (31.00)	53.00 (31.00)	58.00 (32.00)	< 0.001
Sirs	3.00 (1.00)	3.00 (1.00)	3.00 (1.00)	0.873
Sapsii	39.00 (19.00)	38.00 (20.00)	41.00 (20.00)	0.041
Oasis	32.00 (12.00)	32.00 (12.00)	34.00 (13.00)	< 0.001
Gcs	15.00 (1.00)	15.00 (1.00)	15.00 (1.00)	0.682
Charlson	6.00 (4.00)	6.00 (4.00)	5.00 (3.00)	0.260
MELD	19.79 (14.04)	19.02 (13.84)	21.88 (14.27)	< 0.001
MELD_NA,	21.44 (14.59)	20.95 (14.46)	23.19 (14.80)	< 0.001
CTP	8.00 (3.00)	8.00 (3.00)	9.00 (3.00)	< 0.001
**Drugs**				
Anticoagulant, n (p%)	2,469.00 (78.13%)	1953.00 (84.51%)	516.00 (60.78%)	< 0.001
Diuretics, n (p%)	1,243.00 (39.34%)	909.00 (39.33%)	334.00 (39.34%)	0.997
GC, n (p%)	1,087.00 (34.40%)	856.00 (37.04%)	231.00 (27.21%)	< 0.001
Abx, n (p%)	2,898.00 (91.71%)	2,076.00 (89.83%)	822.00 (96.82%)	< 0.001
APT, n (p%)	770.00 (24.37%)	647.00 (28.00%)	123.00 (14.49%)	< 0.001
ACand APT, n (p%)	699.00 (22.12%)	599.00 (25.92%)	100.00 (11.78%)	< 0.001

HGB, Hemoglobin; PLT, Platelet; RDW, Red Cell Distribution Width; RBC, Red Blood Cell; WBC, White Blood Cell; ALT, Alanine Aminotransferase; AST, Aspartate Aminotransferase; TBIL, Total Bilirubin; BUN, Blood Urea Nitrogen; CRE, Creatinine; INR, International Normalized Ratio; PT, Prothrombin Time; HE, Hepatic Encephalopathy; HTN, Hypertension; AKI, Acute Kidney Injury; CKD, Chronic Kidney Disease; IHD, Ischemic Heart Disease; COPD, Chronic Obstructive Pulmonary Disease; SOFA, Sequential Organ Failure Assessment; APSIII, Acute Physiology Score III; SAPSII, Simplified Acute Physiological Score II; OASIS, Oxford Acute Severity of Illness Score; GCS, Glasgow Coma Scale; MELD, Model for End-Stage Liver Disease; MELD-Na Model for End-Stage Liver Disease-Sodium; CTP, Child–Turcotte–Pugh; GC, Glasgow Coma; ABX, Antibiotics; APT, Antiplatelet Therapy; AC, Anticoagulant.

### Boruta-based identification of risk factors for GIB during ICU stay in cirrhotic patients

**FIGURE 2:**
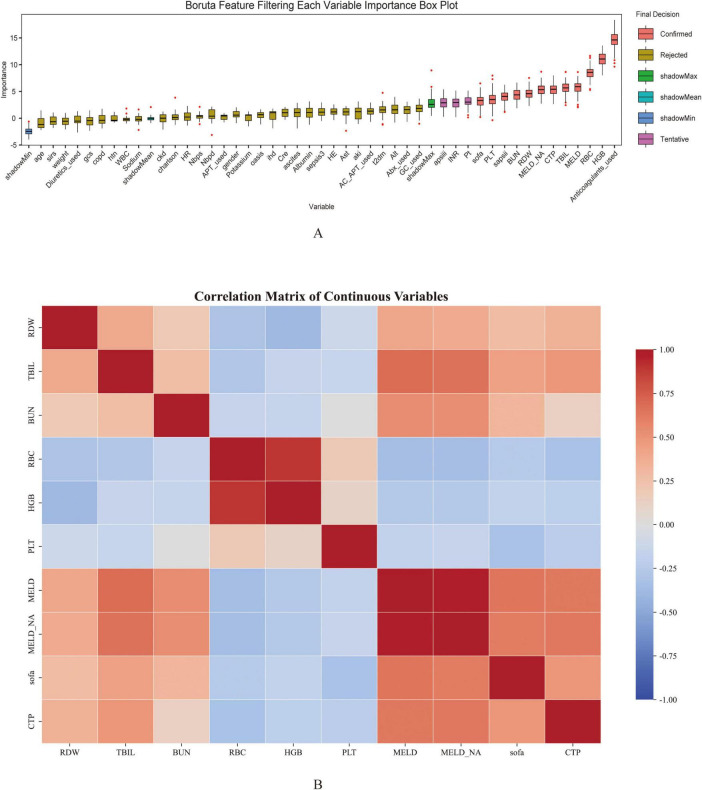
Feature selection using Boruta algorithm **(A)** and the correlation heatmap **(B)** for predicting gastrointestinal bleeding in cirrhosis patients admitted to the ICU. The horizontal axis represents the name of each variable, while the vertical axis represents the Z value of each variable. The box plot illustrates the distribution of Z values during model calculation. Peach pink boxes indicate important variables, yellow boxes indicate unimportant variables, and pink boxes indicate potentially important variables.

To determine the most informative predictors of GIB after ICU admission in patients with cirrhosis, the Boruta algorithm was employed. The Z-score ranking of features, presented in Figure 2A, facilitated the exclusion of irrelevant variables and improved downstream model performance. A total of 12 variables—anticoagulant use, HGB, PLT, RDW, RBC, Sapsii, Sofa, TBIL, BUN, MELD, MELD-Na, and CTP—were identified as having high predictive value and were subsequently incorporated into the model as primary features.

Among the 12 variables selected by the Boruta algorithm, a correlation heatmap was first constructed to explore potential collinearity among continuous variables as shown in Figure 2B. The results indicated a strong correlation between MELD and MELD-Na, suggesting a potential issue of multicollinearity. To further assess this, the VIF was calculated for each variable. Both MELD and MELD-Na demonstrated VIF values exceeding 10, confirming the presence of severe multicollinearity. Based on clinical relevance and their respective associations with the outcome variable, the MELD variable was excluded. After exclusion, VIFs were recalculated, and all remaining variables exhibited VIF values below 10, indicating that multicollinearity had been effectively mitigated. The refined variable set was therefore considered appropriate for subsequent modeling analysis.

### RF demonstrated the highest discriminative ability among evaluated ML algorithms for predicting in-ICU GIB in cirrhosis patients

**FIGURE 3:**
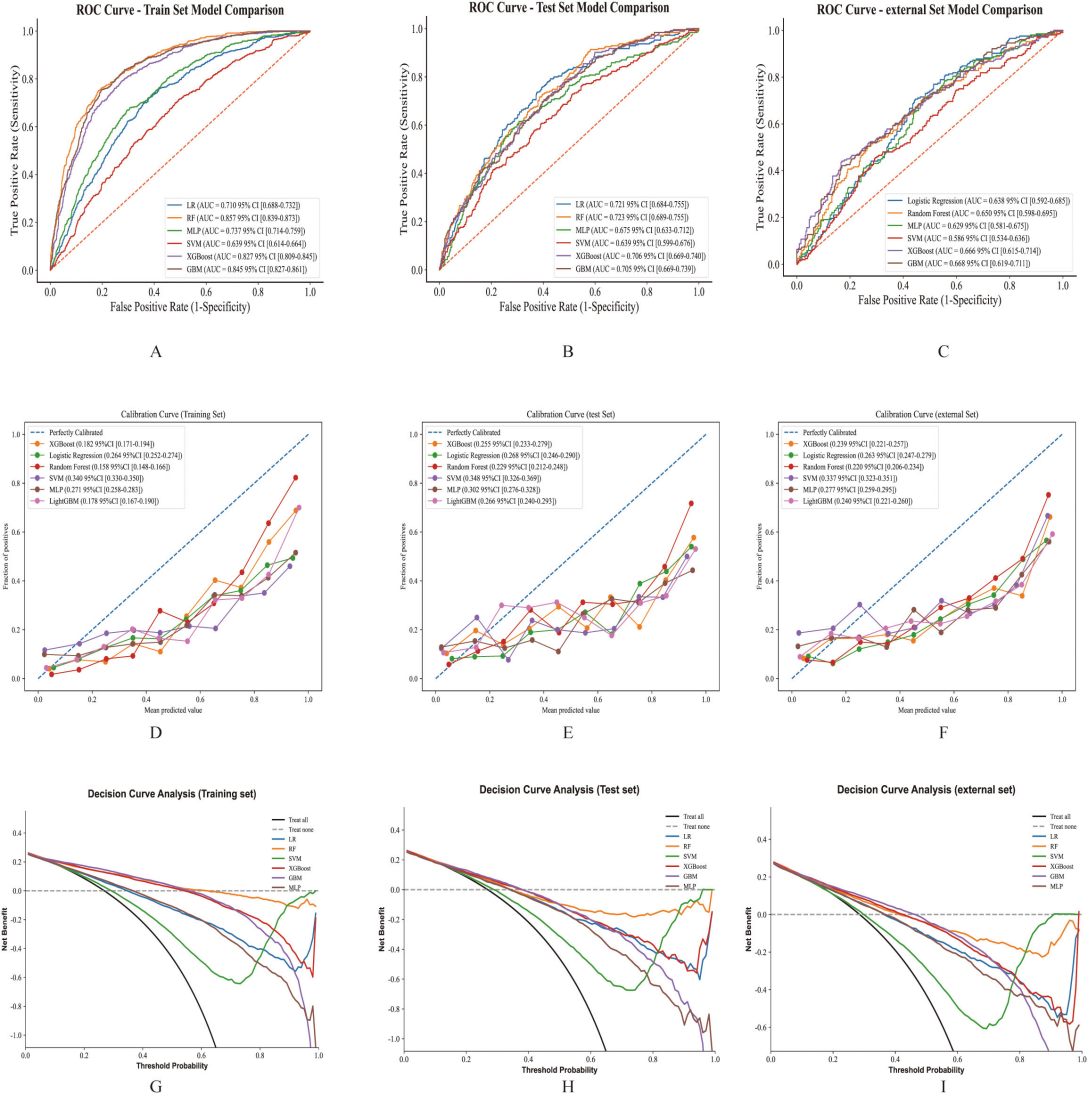
The performance and comparison of six different predictive models. **(A)**. ROC curves for the training set. **(B)** ROC curves for the test set. **(C)** ROC curves for the validation set. **(D)** Calibration curve for the training set. **(E)** Calibration curve for the test set. **(F)** Calibration curve for the validation set. **(G)** Decision curve analysis for the training set. **(H)** Decision curve analysis for the test set. **(I)** Decision curve analysis for the validation set.

In this study, six ML algorithms were implemented to predict GIB occurrence among ICU-admitted patients with cirrhosis. Model performance was evaluated using ROC curves, calibration curves, and DCA, as presented in Figure 3. Across the training, testing, and validation cohorts, the RF model exhibited superior predictive performance and stability. Specifically, the RF model demonstrated strong discriminative ability in the training cohort, achieving an area under the ROC curve (AUROC) of 0.857 (95% CI, 0.839–0.873), with an accuracy of 70.12%, sensitivity of 88.22%, specificity of 63.47%, precision of 47%, and an F1-score of 61.32%. In the test cohort, the RF model maintained relatively robust performance, showing an AUROC of 0.723 (95% CI, 0.689–0.755), accuracy of 59.07%, sensitivity of 76.47%, specificity of 52.67%, precision of 37.28%, and an F1-score of 50.13%, indicating good generalizability.

Compared to other ML models, RF exhibited the highest AUROC in the training cohort and the test cohort (Figures 3A,B). Although GBM displayed the highest AUROC in the external cohort (Figure 3C), GBM was excluded from further consideration due to concerns about potential overfitting and diminished predictive capability in new datasets. Other models, including LR, XGBoost, and MLP, also showed strong performance, yet RF was selected as the optimal model due to its consistent robustness across all datasets (Table 2 and Supplementary Table 4).

**TABLE 2:** Comprehensive performance metrics of multiple machine learning models for predicting GIB in cirrhosis patients admitted to the ICU evaluated across training and test datasets.

		RF	SVM	LGBM	LR	XGBoost	MLP
Training	Accuracy	70.12%	45.57%	73.42%	58.68%	70.16%	58.32%
	Sensitivity	88.22%	86.03%	84.85%	78.45%	84.18%	84.51%
	Precision	47%	31.31%	50.30%	37.22%	46.90%	37.69%
	Specificity	63.47%	30.72%	69.22%	51.45%	65.02%	48.70%
	F1 score	61.32%	45.91%	63.16%	50.49%	60.24%	52.13%
	AUC	85.69%	63.95%	84.47%	70.99%	82.71%	73.74%
Test	Accuracy	59.07%	44.62%	60.86%	60.34%	60.02%	55.06%
	Sensitivity	76.47%	83.92%	72.16%	81.57%	74.12%	78.04%
	Precision	37.28%	30.66%	38.02%	38.73%	37.65%	34.97%
	Specificity	52.67%	30.16%	56.71%	52.53%	54.83%	46.61%
	F1 score	50.13%	44.91%	49.80%	52.53%	49.93%	48.30%
	AUC	72.30%	63.89%	70.46%	72.11%	70.62%	67.55%

RF, Random Forest; SVM, Support Vector Machine; LGBM, Light Gradient Boosting Machine; LR, Logistic Regression; XGBoost, Extreme Gradient Boosting; MLP, Multilayer Perceptron.

### SHAP analysis quantifies feature contributions to In-ICU GIB risk in patients with cirrhosis

**FIGURE 4:**
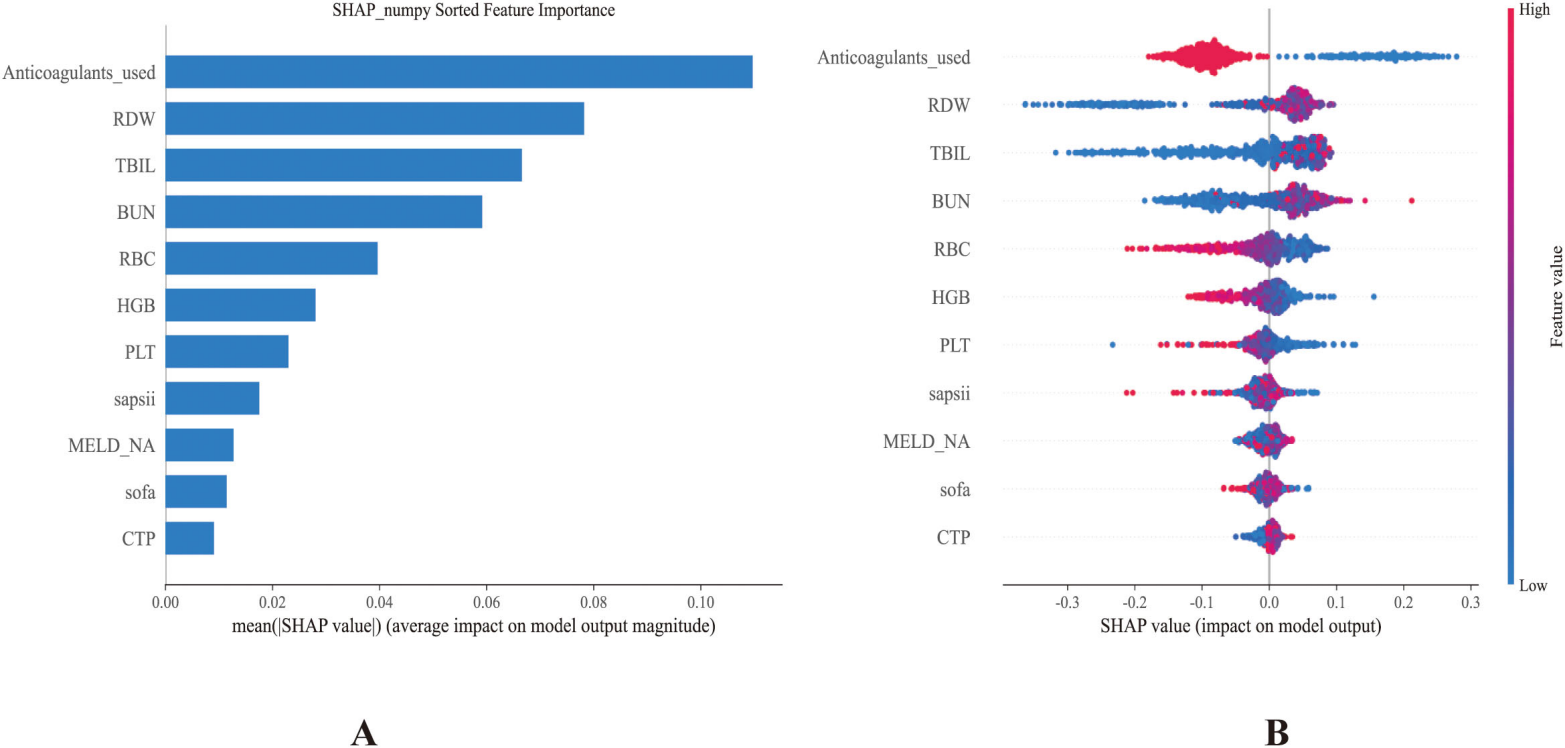
**(A)** Bar plot showing the ranked importance of features in the RF model, based on their average absolute SHAP values. **(B)** Summary plot illustrating the contribution of each feature to the model output. The x-axis represents SHAP values, with each dot corresponding to an individual instance. Red dots indicate higher feature values, while blue dots denote lower ones.

The SHAP method was applied to interpret the prediction results derived from the best-performing RF model. This analysis identified and ranked the 11 most influential features affecting the prediction of GIB risk among ICU-admitted cirrhosis patients, as shown by their SHAP values (Figure 4A). The X-axis (SHAP value) indicates that a higher SHAP value suggests a greater positive impact of the feature on the prediction outcome (i.e., increasing the likelihood of the predicted event), while a lower value signifies that the feature reduces the likelihood of the event. The color gradient (feature value) from red to blue represents the magnitude of the feature value, with red denoting a high feature value and blue indicating a low feature value.

Anticoagulant use was identified as the most influential variable. Higher SHAP values (corresponding to anticoagulant administration) were associated with lower predicted GIB risk, consistent with subsequent regression analysis showing a protective association (OR 0.29, 95% CI 0.24–0.34). Other crucial predictors included RDW, RBC, and PLT. Notably, TBIL and BUN also showed substantial contributions to model predictions. Additional influential variables encompassed higher scores of SAPSII, MELD-Na, SOFA, and CTP. Collectively, these features illustrated their complex and multidimensional relationships within the predictive framework (Figure 4B). For interpretability, individual SHAP value plots (Supplementary Figure 2) were constructed to examine how specific variables contributed to GIB.

These features offered valuable insights into the underlying drivers of GIB in patients with cirrhosis, contributing significantly to both risk prediction and the interpretation of potential pathophysiological mechanisms. The SHAP analysis highlighted anticoagulant use as the strongest predictor, with its administration associated with reduced GIB risk. This paradoxical protective effect may reflect prevention of portal vein thrombosis, maintenance of mesenteric perfusion, or cautious prescribing practices in which anticoagulants are initiated only after careful bleeding risk assessment.

### Kaplan-Meier analysis shows significant impact of GIB on short-term survival in patients with cirrhosis

A Kaplan-Meier survival analysis was conducted to evaluate the impact of GIB on short-term survival in patients with cirrhosis. Supplementary Figure 3 presents the survival curves for patients with and without GIB at 15 and 30 days. The results showed that overall survival was lower in the GIB group than in the non-GIB group, and these differences were statistically significant (*p* = 0.00062 for 15-day survival; *p* < 0.0001 for 30-day survival). These findings suggest that the occurrence of GIB significantly reduced short-term survival in patients with cirrhosis.

### Anticoagulant use identified as a significant predictor of In-ICU GIB risk in patients with cirrhosis

Multivariable logistic regression was performed to further evaluate the association between Anticoagulant use and the risk of in-ICU GIB among patients with cirrhosis. The results, summarized in Table 3, demonstrated a significant relationship between anticoagulant use and GIB occurrence. In the crude, unadjusted analysis, anticoagulants use was significantly associated with decreased odds of GIB (OR 0.29, 95% CI 0.24–0.34, *P* < 0.001). This significant association persisted after sequential adjustments: Model 1 (adjusted for age, gender, and weight), Model 2 (further adjusted for HTN, diabetes, IHD, COPD, sepsis3, and ascites), and Model 3 (additionally adjusted for all laboratory parameters). Across these adjusted models, the OR values remained consistently significant (*P* < 0.001), reinforcing anticoagulant use as a critical predictor of GIB during ICU admission among cirrhotic patients.

**TABLE 3:** Association between anticoagulant therapy and risk of gastrointestinal bleeding in patients with cirrhosis in ICU.

Varoable	OR^a^ (95% CI)	*P–*value
Crude model^b^	0.29 (0.24–0.34)	< 0.001
Model 1^c^	0.29 (0.24–0.35)	< 0.001
Model 2^d^	0.27 (0.22–0.32)	< 0.001
Model 3^e^	0.29 (0.24–0.36)	< 0.001

^a^OR odds ratio.

^b^Crude model: No covariates were adjusted.

^c^Model 1: Adjusted for age, gender, and weight.

^d^Model 2: Model 1 and adjusted for HTN, diabetes, IHD, COPD, sepsis3, and ascites.

^e^Model 3: Model 2 and adjusted for all laboratory parameters.

A Kaplan-Meier analysis was also performed to evaluate the effect of anticoagulant therapy in all patients with cirrhosis. As shown in Supplementary Figure 4, there was no significant difference in mortality at 15 and 30 days (*p* = 0.12 and *p* = 0.99, respectively). However, among cirrhotic patients with gastrointestinal bleeding, the results differed markedly. In this subgroup, those who received anticoagulant therapy had a lower short-term risk of death compared with those who did not, with statistical significance at both 15 and 30 days (*p* = 0.0062 and *p* < 0.001, as shown in Supplementary Figure 5).

**FIGURE 5:**
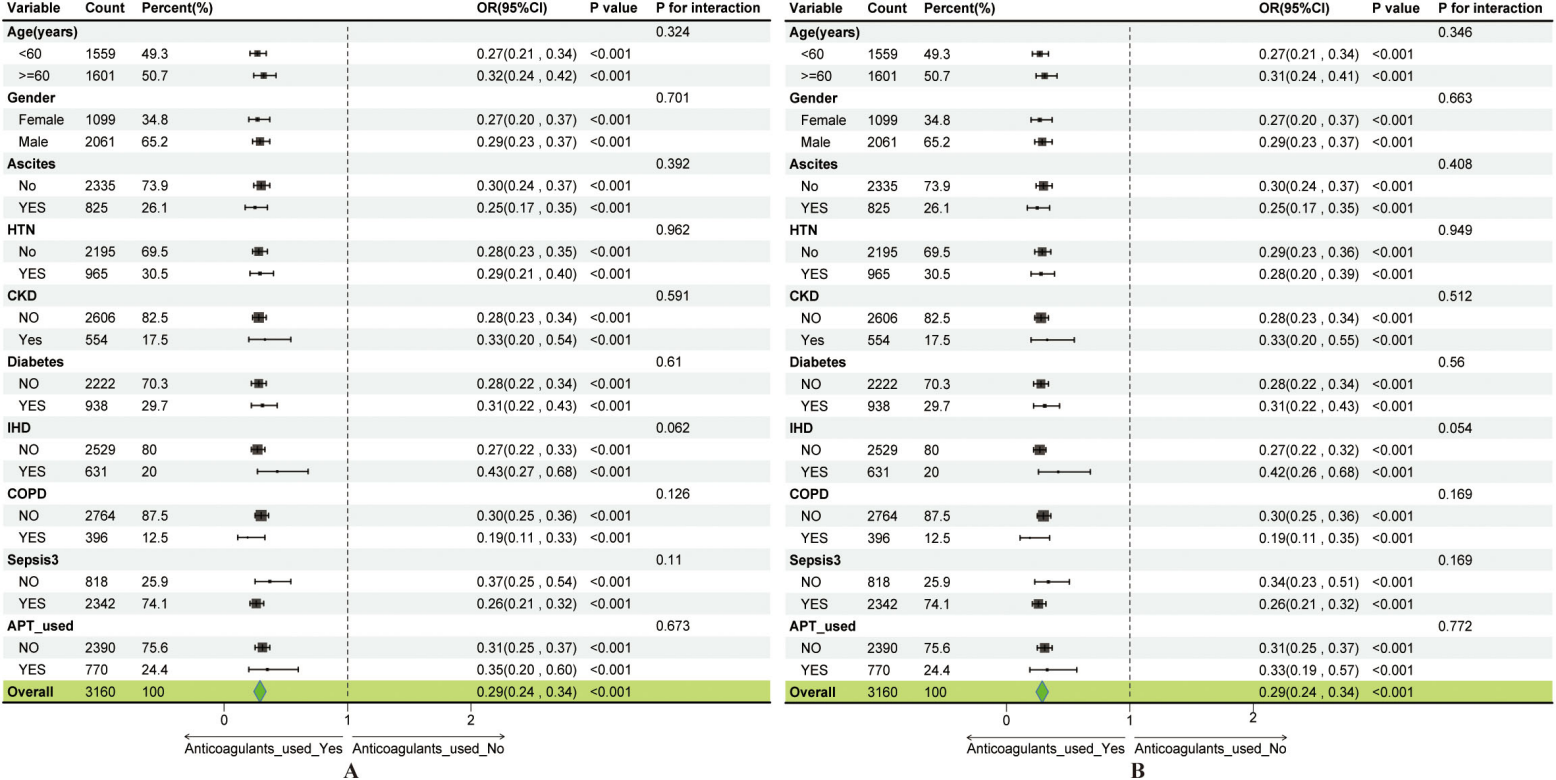
Subgroup analysis showing the association between anticoagulant use and gastrointestinal bleeding. **(A)** Subgroup analysis of the anticoagulant use–GIB association before covariate adjustment. **(B)** Subgroup analysis of the anticoagulant use–GIB association after covariate adjustment.

Moreover, subgroup analyses and interaction tests were conducted using multivariable logistic regression to explore potential modifying effects of clinical and demographic characteristics on the anticoagulant use–GIB association (Figure 5A). Stratified analyses by age, gender, ascites, HTN, CKD, IHD, and other subgroups consistently showed robust associations, indicating that the relationship between anticoagulant use and GIB remained stable across all evaluated subgroups, even after adjustment for covariates (Figure 5B). These findings underscore the generalizable role of anticoagulant use as a significant predictor of GIB risk among cirrhotic patients admitted to the ICU.

## Discussion

This study comprehensively investigates risk factors contributing to GIB among ICU-admitted patients with cirrhosis. Leveraging the Boruta algorithm, key predictive features were identified and evaluated across multiple ML models, ultimately resulting in the development of a robust RF-based predictive model. These findings deepen the understanding of GIB pathophysiology in cirrhotic patients and provide clinicians with a practical tool for early risk stratification to reduce complications during critical care.

Compared to previous studies, this investigation offers several distinct contributions. Notably, patients who experienced GIB demonstrated significantly lower levels of RBC, hemoglobin, and multiple liver function markers compared to those without bleeding events, which is consistent with findings reported in earlier studies (19, 20). For example, Abraldes et al. (21) also observed that even small varices in patients with decompensated cirrhosis carry a measurable risk of hemorrhage, underscoring the clinical relevance of early hematologic and hepatic dysfunction in predicting bleeding episodes. Furthermore, liver function scores including MELD, MELD-Na, and CTP were also significantly associated with the occurrence of GBI, reinforcing prior evidence that impaired hepatic reserve plays a key role in bleeding risk among cirrhotic patients (9). This study reinforces the significance of identified risk factors through multivariable analysis and further improves predictive performance by employing ML techniques. The SOFA score has been widely used and has played a significant role in prognostic assessment of critically ill cirrhotic patients, serving as an established tool for evaluating organ dysfunction and predicting clinical outcomes in intensive care settings (22). While the SOFA score yielded AUCs of 0.550 in the training set and 0.579 in the test set, our RF model achieved substantially higher AUCs of 0.857 (training) and 0.723 (test), indicating markedly improved predictive accuracy. Unlike previous research that primarily focused on upper gastrointestinal bleeding alone (11), the present analysis encompasses a broader range of GIB cases, involves a relatively larger patient cohort, and integrates external validation to assess the stability and generalizability of predictive models. Additionally, utilizing the Boruta algorithm for feature selection combined with the implementation of the RF model contributes to enhanced robustness and achieves superior predictive accuracy, as demonstrated by an AUC of 0.723 in the text set. These methodological advancements highlight the potential of ML-based approaches for precise risk stratification, particularly in the context of complex, high-dimensional clinical datasets.

Moreover, the association between anticoagulant therapy and GIB remained statistically significant in multivariable logistic regression analyses, even after controlling for potential confounders. Anticoagulant use was associated with lower GIB risk—a finding that contrasts with conventional expectations but aligns with emerging evidence on rebalanced hemostasis in cirrhosis (23). This unexpected protective association may be partly attributable to cautious prescribing practices in ICU settings, where anticoagulants are typically administered only after rigorous evaluation, explicitly including bleeding risk assessment. Although patients with liver cirrhosis have historically been characterized by an increased propensity toward bleeding, recent evidence has introduced the concept of “rebalanced hemostasis.” Under these conditions, marked reductions occur in both procoagulant and anticoagulant factors, including substantial decreases in endogenous anticoagulants such as proteins C and S, as well as antithrombin III, potentially tipping the balance toward a prothrombotic state (24). Consequently, anticoagulation could confer protective effects by preventing mesenteric or portal vein thrombosis, mitigating local ischemia, and reducing secondary bleeding episodes. Moreover, anticoagulants such as low-molecular-weight heparin have demonstrated efficacy in preventing portal vein thrombosis (PVT), a crucial factor contributing to worsening portal hypertension and a known exacerbator of gastroesophageal variceal hemorrhage. By preventing or controlling PVT, anticoagulation therapy may indirectly diminish the likelihood of variceal rupture, thereby decreasing overall GIB incidence (25). Additionally, intestinal hypoperfusion frequently occurs in critically ill patients, predisposing them to mucosal ischemia. Here, anticoagulants might exert beneficial effects by inhibiting microthrombus formation, enhancing microcirculatory perfusion, and ultimately reducing the occurrence of stress-induced mucosal injury and related gastrointestinal bleeding (26).

This study presents a novel application of machine learning algorithms to predict GIB in patients with cirrhosis by integrating comprehensive clinical and laboratory data. The use of the MIMIC-IV database, alongside external validation with the EICU dataset, strengthens the robustness and generalizability of the findings across heterogeneous patient populations. Nevertheless, several limitations of this study should be acknowledged. First, the analysis was conducted using the MIMIC-IV database, which is a large, publicly available, single-center critical care dataset. While this dataset provides valuable insights, it is important to note that our study is retrospective in design, which may introduce inherent biases related to documentation and patient selection. Although we performed external validation using the EICU database to strengthen the generalizability of our model, the retrospective nature of both datasets still poses potential limitations in terms of temporal bias and the inability to capture real-time clinical decisions. Future prospective studies are needed to validate our findings in a more dynamic, real-world clinical setting. Second, the absence of granular information regarding surgical or interventional procedures may have introduced unmeasured confounding, as such interventions may impact both bleeding risk and clinical trajectories. Third, GIB events were identified using ICD diagnostic codes without corroboration from endoscopy reports, transfusion records, or hemoglobin trends. While this approach facilitates reproducibility and enables validation across large databases, it may introduce misclassification. Future prospective studies incorporating multi-modal event validation would enhance outcome accuracy. Fourth, due to the retrospective nature of our data and challenges in accurately determining GIB onset timing from electronic health records, we could not reliably exclude patients whose bleeding may have started within the first 24 h after admission. This limitation may introduce minimal time-dependent confounding, as early medication exposures could be influenced by concurrent bleeding in some patients. However, this reflects practical constraints of administrative data and the clinical reality that early ICU risk stratification often occurs when bleeding risk is already developing. Future prospective studies with real-time bleeding monitoring and standardized event adjudication could use stricter landmark designs to reduce temporal bias. At last, while the study specifically addressed the incidence of GIB among ICU patients with liver cirrhosis, it did not evaluate downstream outcomes. Future research should aim to elucidate the long-term prognostic implications of GIB in this population, including mortality, ICU and hospital length of stay, and the risk of recurrent bleeding.

## Conclusion

The RF model achieved moderate-to-good discrimination for predicting in-ICU GIB among patients with cirrhosis, with anticoagulant use, hemoglobin, and platelet count identified as key predictors. Prospective validation is needed to confirm the model’s clinical utility in guiding preventive interventions and to further elucidate the protective association observed with anticoagulant therapy.

## Data Availability

The raw data supporting the conclusions of this article will be made available by the authors, without undue reservation.
