# Supplementary material for: Machine learning–based risk stratification for gastrointestinal bleeding in ICU patients with cirrhosis: evidence from the MIMIC database
Source: Front Med (Lausanne). 2025 Dec 5;12:1701973. doi: 10.3389/fmed.2025.1701973 (PMC12714975; doi:10.3389/fmed.2025.1701973)
Supplement: Supplementary file 1 [file Data_Sheet_1.docx]

**Supplementary Materials**

**Table S1** **Summary of Variables with Missing Values**

| Variable | Count |
| --- | --- |
| D.Dimer | 0.976265823 |
| NT_proBNP | 0.922784810 |
| Protein_total | 0.896202532 |
| CK | 0.634493671 |
| IBIL | 0.622784810 |
| NEUC | 0.612658228 |
| DBIL | 0.596835443 |
| height | 0.427531646 |
| Albumin | 0.137658228 |
| Alt | 0.052215190 |
| Ast | 0.045253165 |
| TBIL | 0.043354430 |
| INR | 0.024050633 |
| Pt | 0.023417722 |
| RDW | 0.011708861 |
| weight | 0.011392405 |
| Nbps | 0.010759494 |
| Nbpd | 0.010759494 |
| PLT | 0.009493671 |
| HGB | 0.008227848 |
| RBC | 0.007911392 |
| WBC | 0.007278481 |
| Potassium | 0.003797468 |
| Cre | 0.003164557 |
| BUN | 0.003164557 |
| Sodium | 0.002848101 |
| gcs | 0.002215190 |

*D.Dimer* **D-dimer**; *NT_proBNP* **N-terminal pro–B-type natriuretic peptide**; *Protein_total* **Total protein**; *CK* **Creatine kinase**; *IBIL* **Indirect bilirubin**; *NEUC* **Neutrophil count**; *DBIL* **Direct bilirubin**; *Alt* **Alanine aminotransferase**; *Ast* **Aspartate aminotransferase**; *TBIL* **Total bilirubin**; *INR* **International normalized ratio**; *Pt* **Prothrombin time**; *RDW* **Red cell distribution;** *Nbps* **Noninvasive systolic blood pressure**; *Nbpd* **Noninvasive diastolic blood pressure**; *PLT* **Platelet**; *HGB* **Hemoglobin**; *RBC* **Red blood cell**; *WBC* **White blood cell**; *Cre* **Creatinine**; *BUN* **Blood urea nitrogen**; *gcs* **Glasgow coma scale**.

**Table S2 Baseline Characteristics of the Training Cohort**

| Variable | Levels | Overall | 0 | 1 | p-value |
| --- | --- | --- | --- | --- | --- |
|  |  | N = 2,528 | N = 1,849 | N = 679 |  |
| age, median (IQR) | | 60.00 (15.00) | 60.00 (15.00) | 58.00 (15.00) | <0.001 |
| weight, median (IQR) | | 82.78 (29.35) | 82.80 (30.45) | 82.60 (27.40) | 0.425 |
| HGB, median (IQR) | | 9.40 (2.70) | 9.60 (2.80) | 8.80 (2.60) | <0.001 |
| PLT, median (IQR) | | 103.00 (93.50) | 107.00 (98.00) | 93.00 (85.00) | <0.001 |
| RDW, median (IQR) | | 16.70 (3.70) | 16.50 (3.70) | 17.40 (3.70) | <0.001 |
| RBC, median (IQR) | | 3.00 (0.97) | 3.08 (0.99) | 2.77 (0.86) | <0.001 |
| WBC, median (IQR) | | 9.60 (8.50) | 9.60 (8.40) | 9.40 (8.60) | 0.261 |
| Albumin, median (IQR) | | 3.00 (0.90) | 3.00 (0.90) | 2.90 (0.80) | 0.003 |
| Potassium, median (IQR) | | 4.20 (1.00) | 4.10 (1.00) | 4.20 (1.20) | 0.001 |
| Sodium, median (IQR) | | 137.00 (7.00) | 137.00 (7.00) | 137.00 (8.00) | 0.146 |
| Alt, median (IQR) | | 33.00 (54.00) | 33.00 (61.00) | 34.00 (43.00) | 0.004 |
| Ast, median (IQR) | | 69.00 (117.00) | 67.00 (118.00) | 75.00 (110.00) | 0.691 |
| TBIL, median (IQR) | | 2.60 (5.60) | 2.30 (5.00) | 3.70 (7.80) | <0.001 |
| Cre, median (IQR) | | 1.20 (1.30) | 1.20 (1.30) | 1.20 (1.30) | 0.885 |
| BUN, median (IQR) | | 26.00 (30.00) | 24.00 (29.00) | 30.00 (33.00) | <0.001 |
| INR, median (IQR) | | 1.70 (0.80) | 1.60 (0.80) | 1.80 (0.80) | <0.001 |
| Pt, median (IQR) | | 18.40 (8.00) | 18.00 (7.90) | 19.20 (8.40) | 0.001 |
| HR, median (IQR) | | 91.00 (27.00) | 90.00 (26.00) | 93.00 (28.00) | 0.072 |
| Nbps, median (IQR) | | 117.00 (33.50) | 117.00 (34.00) | 116.00 (32.00) | 0.290 |
| Nbpd, median (IQR) | | 66.00 (22.00) | 67.00 (23.00) | 64.00 (22.00) | 0.104 |
| sofa, median (IQR) | | 8.00 (6.00) | 8.00 (6.00) | 8.00 (6.00) | <0.001 |
| apsiii, median (IQR) | | 54.50 (31.00) | 54.00 (31.00) | 57.00 (34.00) | <0.001 |
| sirs, median (IQR) | | 3.00 (1.00) | 3.00 (1.00) | 3.00 (1.00) | 0.881 |
| sapsii, median (IQR) | | 39.00 (19.00) | 38.00 (19.00) | 40.00 (20.00) | 0.138 |
| oasis, median (IQR) | | 32.00 (12.00) | 32.00 (12.00) | 33.00 (13.00) | 0.003 |
| gcs, median (IQR) | | 15.00 (1.00) | 15.00 (1.00) | 15.00 (1.00) | 0.870 |
| charlson, median (IQR) | | 6.00 (4.00) | 6.00 (4.00) | 5.00 (3.00) | 0.760 |
| MELD, median (IQR) | | 19.94 (13.99) | 19.13 (13.72) | 22.02 (14.31) | <0.001 |
| MELD_NA, median (IQR) | | 21.69 (14.49) | 21.17 (14.26) | 23.24 (14.79) | <0.001 |
| CTP, median (IQR) | | 8.00 (3.00) | 8.00 (3.00) | 9.00 (3.00) | <0.001 |
| gender, n (p%) |  |  |  |  | 0.794 |
|  | 0 | 889.00 (35.17%) | 653.00 (35.32%) | 236.00 (34.76%) |  |
|  | 1 | 1,639.00 (64.83%) | 1,196.00 (64.68%) | 443.00 (65.24%) |  |
| ascites, n (p%) |  |  |  |  | <0.001 |
|  | 0 | 1,862.00 (73.66%) | 1,396.00 (75.50%) | 466.00 (68.63%) |  |
|  | 1 | 666.00 (26.34%) | 453.00 (24.50%) | 213.00 (31.37%) |  |
| htn, n (p%) |  |  |  |  | 0.462 |
|  | 0 | 1,763.00 (69.74%) | 1,297.00 (70.15%) | 466.00 (68.63%) |  |
|  | 1 | 765.00 (30.26%) | 552.00 (29.85%) | 213.00 (31.37%) |  |
| aki, n (p%) |  |  |  |  | 0.101 |
|  | 0 | 1,065.00 (42.13%) | 797.00 (43.10%) | 268.00 (39.47%) |  |
|  | 1 | 1,463.00 (57.87%) | 1,052.00 (56.90%) | 411.00 (60.53%) |  |
| ckd, n (p%) |  |  |  |  | 0.175 |
|  | 0 | 2,083.00 (82.40%) | 1,512.00 (81.77%) | 571.00 (84.09%) |  |
|  | 1 | 445.00 (17.60%) | 337.00 (18.23%) | 108.00 (15.91%) |  |
| Diabetes, n (p%) |  |  |  |  | 0.197 |
|  | 0 | 1,783.00 (70.53%) | 1,291.00 (69.82%) | 492.00 (72.46%) |  |
|  | 1 | 745.00 (29.47%) | 558.00 (30.18%) | 187.00 (27.54%) |  |
| Ihd, n (p%) |  |  |  |  | 0.039 |
|  | 0 | 2,016.00 (79.75%) | 1,456.00 (78.75%) | 560.00 (82.47%) |  |
|  | 1 | 512.00 (20.25%) | 393.00 (21.25%) | 119.00 (17.53%) |  |
| COPD, n (p%) |  |  |  |  | 0.044 |
|  | 0 | 2,212.00 (87.50%) | 1,603.00 (86.70%) | 609.00 (89.69%) |  |
|  | 1 | 316.00 (12.50%) | 246.00 (13.30%) | 70.00 (10.31%) |  |
| HE, n (p%) |  |  |  |  | <0.001 |
|  | 0 | 2,304.00 (91.14%) | 1,717.00 (92.86%) | 587.00 (86.45%) |  |
|  | 1 | 224.00 (8.86%) | 132.00 (7.14%) | 92.00 (13.55%) |  |
| sepsis3, n (p%) |  |  |  |  | <0.001 |
|  | 0 | 668.00 (26.42%) | 529.00 (28.61%) | 139.00 (20.47%) |  |
|  | 1 | 1,860.00 (73.58%) | 1,320.00 (71.39%) | 540.00 (79.53%) |  |
| Anticoagulants_used, n (p%) | |  |  |  | <0.001 |
|  | 0 | 542 (21.44%) | 284.00 (15.36%) | 258.00 (38%) |  |
|  | 1 | 1986(78.56%) | 1,565.00 (84.64%) | 421.00 (62%) |  |
| Diuretics_used, n (p%) | |  |  |  | 0.825 |
|  | 0 | 1,528.00 (60.44%) | 1,120.00 (60.57%) | 408.00 (60.09%) |  |
|  | 1 | 1,000.00 (39.56%) | 729.00 (39.43%) | 271.00 (39.91%) |  |
| GC_used, n (p%) | |  |  |  | <0.001 |
|  | 0 | 1,653.00 (65.39%) | 1,161.00 (62.79%) | 492.00 (72.46%) |  |
|  | 1 | 875.00 (34.61%) | 688.00 (37.21%) | 187.00 (27.54%) |  |
| Abx_used, n (p%) | |  |  |  | <0.001 |
|  | 0 | 211.00 (8.35%) | 187.00 (10.11%) | 24.00 (3.53%) |  |
|  | 1 | 2,317.00 (91.65%) | 1,662.00 (89.89%) | 655.00 (96.47%) |  |
| APT_used, n (p%) | |  |  |  | <0.001 |
|  | 0 | 1,906.00 (75.40%) | 1,327.00 (71.77%) | 579.00 (85.27%) |  |
|  | 1 | 622.00 (24.60%) | 522.00 (28.23%) | 100.00 (14.73%) |  |
| AC_APT_used, n (p%) | |  |  |  | <0.001 |
|  | 0 | 1,967.00 (77.81%) | 1,368.00 (73.99%) | 599.00 (88.22%) |  |
|  | 1 | 561.00 (22.19%) | 481.00 (26.01%) | 80.00 (11.78%) |  |

*HGB* **Hemoglobin;** *PLT* **Platelet;** *RDW* **Red cell distribution width;** *RBC* **Red blood cell;**

*WBC* **White blood cell;** *Alt* **Alanine aminotransferase;** *Ast* **Aspartate aminotransferase;** *TBIL* **Total bilirubin;** *BUN* **Blood urea nitrogen;** *Cre* **Creatinine;** *INR* **International normalized ratio;** *Pt* **Prothrombin time;** *HE* **Hepatic encephalopathy;** *HTN* **Hypertension;** *AKI* **Acute kidney injury;** *CKD* **Chronic kidney disease;** *IHD* **Ischemic heart disease;** *COPD* **Chronic obstructive pulmonary disease;** *SOFA* **Sequential organ failure assessment;** *APSIII*, **Acute physiology score III;** *SAPSII*, **Simplifed acute physiological score** II; *OASIS*, **oxford acute severity of illness score;** *GCS*, **Glasgow coma scale;** *MELD* **Model for end-stage liver disease;** **MELD-Na Model for end-stage liver disease-sodium;** *CTP* **Child–Turcotte–Pugh;** *GC* **Glasgow coma;** *Abx* **Antibiotics**; *APT* **Antiplatelet therapy;** *AC* **Anticoagulant**

**Table S3** **Baseline Characteristics of the Text Cohort**

| Variable | Levels | Overall | 0 | 1 | p-value |
| --- | --- | --- | --- | --- | --- |
|  |  | N = 632 | N = 462 | N = 170 |  |
| age, median (IQR) | | 59.00 (15.00) | 60.00 (15.00) | 57.00 (14.00) | 0.005 |
| weight, median (IQR) | | 81.85 (29.27) | 82.00 (29.30) | 81.78 (27.00) | 0.722 |
| HGB, median (IQR) | | 9.40 (2.80) | 9.60 (2.70) | 8.90 (2.70) | <0.001 |
| PLT, median (IQR) | | 100.00 (84.00) | 102.00 (86.00) | 91.00 (72.00) | 0.054 |
| RDW, median (IQR) | | 16.70 (4.05) | 16.00 (4.10) | 17.80 (3.40) | <0.001 |
| RBC, median (IQR) | | 2.99 (0.94) | 3.07 (0.97) | 2.87 (0.92) | <0.001 |
| WBC, median (IQR) | | 9.25 (8.55) | 9.10 (8.80) | 9.80 (7.60) | 0.320 |
| Albumin, median (IQR) | | 2.90 (0.90) | 3.00 (0.80) | 2.80 (0.70) | 0.018 |
| Potassium, median (IQR) | | 4.20 (1.00) | 4.10 (0.90) | 4.40 (1.20) | 0.007 |
| Sodium, median (IQR) | | 137.00 (8.00) | 137.00 (7.00) | 138.00 (8.00) | 0.429 |
| Alt, median (IQR) | | 33.00 (47.50) | 34.00 (57.00) | 31.00 (29.00) | 0.006 |
| Ast, median (IQR) | | 66.50 (113.00) | 65.50 (121.00) | 69.00 (96.00) | 0.645 |
| TBIL, median (IQR) | | 2.60 (5.85) | 2.40 (4.90) | 3.30 (7.50) | 0.005 |
| Cre, median (IQR) | | 1.10 (1.30) | 1.10 (1.30) | 1.20 (1.60) | 0.219 |
| BUN, median (IQR) | | 24.00 (27.00) | 22.50 (27.00) | 28.50 (29.00) | 0.102 |
| INR, median (IQR) | | 1.65 (0.70) | 1.60 (0.80) | 1.80 (0.80) | 0.092 |
| Pt, median (IQR) | | 18.05 (7.70) | 17.45 (7.40) | 19.10 (8.30) | 0.072 |
| HR, median (IQR) | | 91.00 (27.00) | 91.00 (26.00) | 90.00 (28.00) | 0.714 |
| Nbps, median (IQR) | | 115.00 (32.00) | 117.00 (35.00) | 113.00 (25.00) | 0.004 |
| Nbpd, median (IQR) | | 65.00 (22.00) | 66.00 (22.00) | 62.00 (23.00) | 0.153 |
| sofa, median (IQR) | | 8.00 (6.00) | 8.00 (6.00) | 10.00 (6.00) | <0.001 |
| apsiii, median (IQR) | | 54.50 (32.00) | 53.00 (34.00) | 61.50 (26.00) | 0.029 |
| sirs, median (IQR) | | 3.00 (1.00) | 3.00 (1.00) | 3.00 (1.00) | 0.525 |
| sapsii, median (IQR) | | 38.00 (21.00) | 37.00 (20.00) | 42.00 (19.00) | 0.103 |
| oasis, median (IQR) | | 32.50 (12.00) | 32.00 (12.00) | 34.00 (12.00) | 0.022 |
| gcs, median (IQR) | | 15.00 (1.00) | 15.00 (2.00) | 15.00 (1.00) | 0.552 |
| charlson, median (IQR) | | 6.00 (4.00) | 6.00 (4.00) | 5.00 (3.00) | 0.047 |
| MELD, median (IQR) | | 19.21 (14.07) | 18.48 (14.13) | 21.34 (14.28) | 0.005 |
| MELD_NA, median (IQR) | | 20.81 (14.96) | 20.37 (15.20) | 23.06 (14.96) | 0.016 |
| CTP, median (IQR) | | 8.00 (3.00) | 8.00 (2.00) | 9.00 (2.00) | <0.001 |
| gender, n (p%) |  |  |  |  | 0.773 |
|  | 0 | 210.00 (33.23%) | 152.00 (32.90%) | 58.00 (34.12%) |  |
|  | 1 | 422.00 (66.77%) | 310.00 (67.10%) | 112.00 (65.88%) |  |
| ascites, n (p%) |  |  |  |  | 0.020 |
|  | 0 | 473.00 (74.84%) | 357.00 (77.27%) | 116.00 (68.24%) |  |
|  | 1 | 159.00 (25.16%) | 105.00 (22.73%) | 54.00 (31.76%) |  |
| htn, n (p%) |  |  |  |  | 0.059 |
|  | 0 | 432.00 (68.35%) | 306.00 (66.23%) | 126.00 (74.12%) |  |
|  | 1 | 200.00 (31.65%) | 156.00 (33.77%) | 44.00 (25.88%) |  |
| aki, n (p%) |  |  |  |  | 0.124 |
|  | 0 | 277.00 (43.83%) | 211.00 (45.67%) | 66.00 (38.82%) |  |
|  | 1 | 355.00 (56.17%) | 251.00 (54.33%) | 104.00 (61.18%) |  |
| ckd, n (p%) |  |  |  |  | 0.582 |
|  | 0 | 523.00 (82.75%) | 380.00 (82.25%) | 143.00 (84.12%) |  |
|  | 1 | 109.00 (17.25%) | 82.00 (17.75%) | 27.00 (15.88%) |  |
| t2dm, n (p%) |  |  |  |  | 0.007 |
|  | 0 | 439.00 (69.46%) | 307.00 (66.45%) | 132.00 (77.65%) |  |
|  | 1 | 193.00 (30.54%) | 155.00 (33.55%) | 38.00 (22.35%) |  |
| ihd, n (p%) |  |  |  |  | 0.108 |
|  | 0 | 513.00 (81.17%) | 368.00 (79.65%) | 145.00 (85.29%) |  |
|  | 1 | 119.00 (18.83%) | 94.00 (20.35%) | 25.00 (14.71%) |  |
| copd, n (p%) |  |  |  |  | 0.223 |
|  | 0 | 552.00 (87.34%) | 399.00 (86.36%) | 153.00 (90.00%) |  |
|  | 1 | 80.00 (12.66%) | 63.00 (13.64%) | 17.00 (10.00%) |  |
| HE, n (p%) |  |  |  |  | 0.014 |
|  | 0 | 557.00 (88.13%) | 416.00 (90.04%) | 141.00 (82.94%) |  |
|  | 1 | 75.00 (11.87%) | 46.00 (9.96%) | 29.00 (17.06%) |  |
| sepsis3, n (p%) |  |  |  |  | <0.001 |
|  | 0 | 150.00 (23.73%) | 128.00 (27.71%) | 22.00 (12.94%) |  |
|  | 1 | 482.00 (76.27%) | 334.00 (72.29%) | 148.00 (87.06%) |  |
| Anticoagulants_used, n (p%) | |  |  |  | <0.001 |
|  | 0 | 149.00 (23.58) | 74.00 (12.02%) | 75.00 (44.12%) |  |
|  | 1 | 483.00 (76.42) | 388.00 (83.98%) | 95.00 (55.88%) |  |
| Diuretics_used, n (p%) | |  |  |  | 0.663 |
|  | 0 | 389.00 (61.55%) | 282.00 (61.04%) | 107.00 (62.94%) |  |
|  | 1 | 243.00 (38.45%) | 180.00 (38.96%) | 63.00 (37.06%) |  |
| GC_used, n (p%) | |  |  |  | 0.013 |
|  | 0 | 420.00 (66.46%) | 294.00 (63.64%) | 126.00 (74.12%) |  |
|  | 1 | 212.00 (33.54%) | 168.00 (36.36%) | 44.00 (25.88%) |  |
| Abx_used, n (p%) | |  |  |  | <0.001 |
|  | 0 | 51.00 (8.07%) | 48.00 (10.39%) | 3.00 (1.76%) |  |
|  | 1 | 581.00 (91.93%) | 414.00 (89.61%) | 167.00 (98.24%) |  |
| APT_used, n (p%) | |  |  |  | <0.001 |
|  | 0 | 484.00 (76.58%) | 337.00 (72.94%) | 147.00 (86.47%) |  |
|  | 1 | 148.00 (23.42%) | 125.00 (27.06%) | 23.00 (13.53%) |  |
| AC_APT_used, n (p%) | |  |  |  | <0.001 |
|  | 0 | 499.00 (78.96%) | 348.00 (75.32%) | 151.00 (88.82%) |  |
|  | 1 | 133.00 (21.04%) | 114.00 (24.68%) | 19.00 (11.18%) |  |

**Table S4** **Comparison of Brier Scores, Calibration-in-the-Large, and Calibration Slope in Training and Testing Cohort**

|  | | RF | SVM | LGBM | LR | XGBoost | MLP |
| --- | --- | --- | --- | --- | --- | --- | --- |
| Training | Brier | 0.158(95%CI 0.148-0.166) | 0.340(95%CI 0.330-0.350) | 0.178(95%CI 0.167-0.190) | 0.264(95%CI 0.252-0.274) | 0.182(95%CI 0.171-0.194) | 0.271(95%CI 0.258-0.283) |
|  | CITL | -1.55 | -1.366 | -1.339 | -1.318 | -1.368 | -1.370 |
|  | Slope | 1.177 | 0.525 | 0.795 | 0.545 | 0.835 | 0.377 |
| Test | Brier | 0.229(95%CI 0.212-0.248) | 0.348(95%CI 0.326-0.369) | 0.266(95%CI 0.240-0.293) | 0.268(95%CI 0.246-0.290) | 0.255(95%CI 0.233-0.279) | 0.302(95%CI 0.276-0.328) |
|  | CITL | -1.179 | -1.365 | -1.083 | -1.347 | -1.124 | -1.230 |
|  | Slope | 0.666 | 0.510 | 0.410 | 0.577 | 0.463 | 0.244 |

*CITL* **Calibration-in-the-Large**


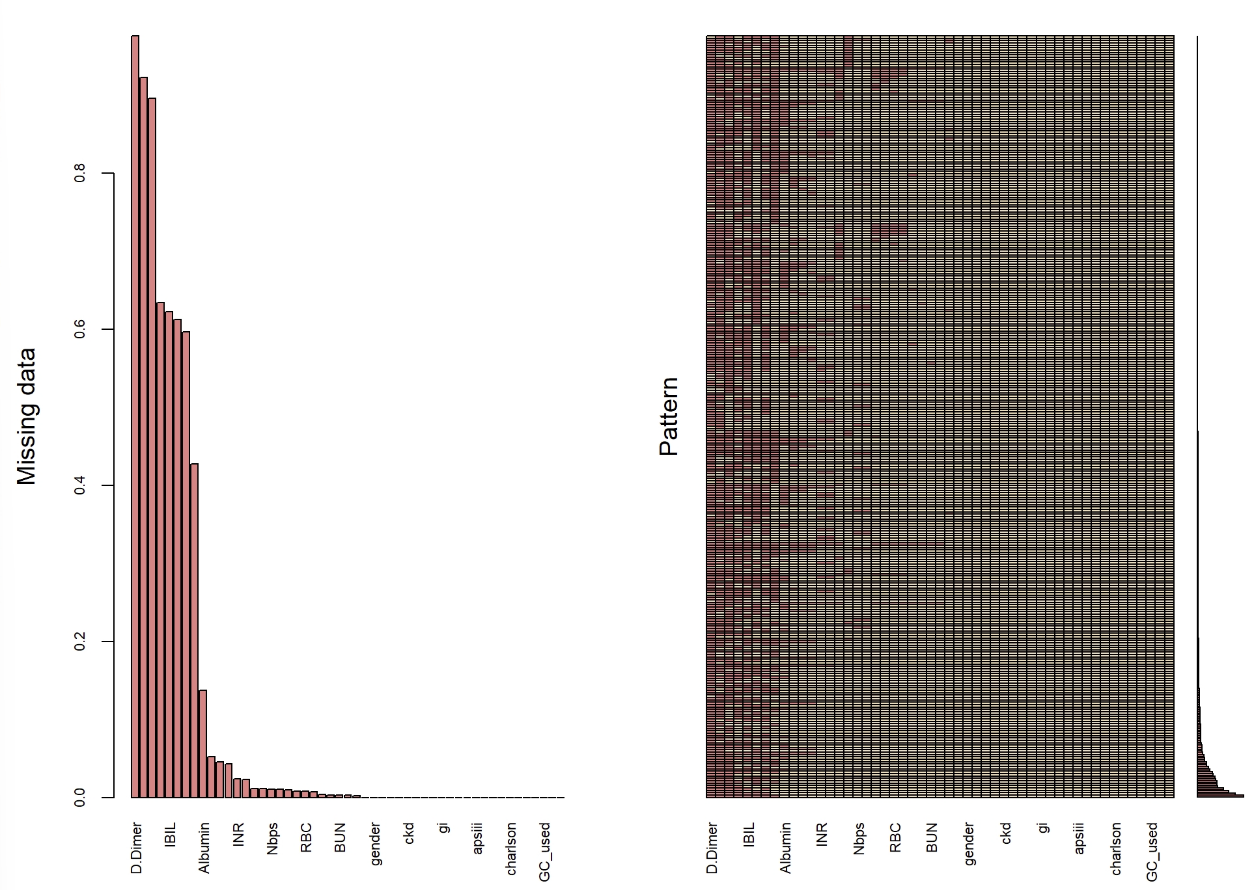


**Fig. S1 Visualization of Missing Data Across Variables**


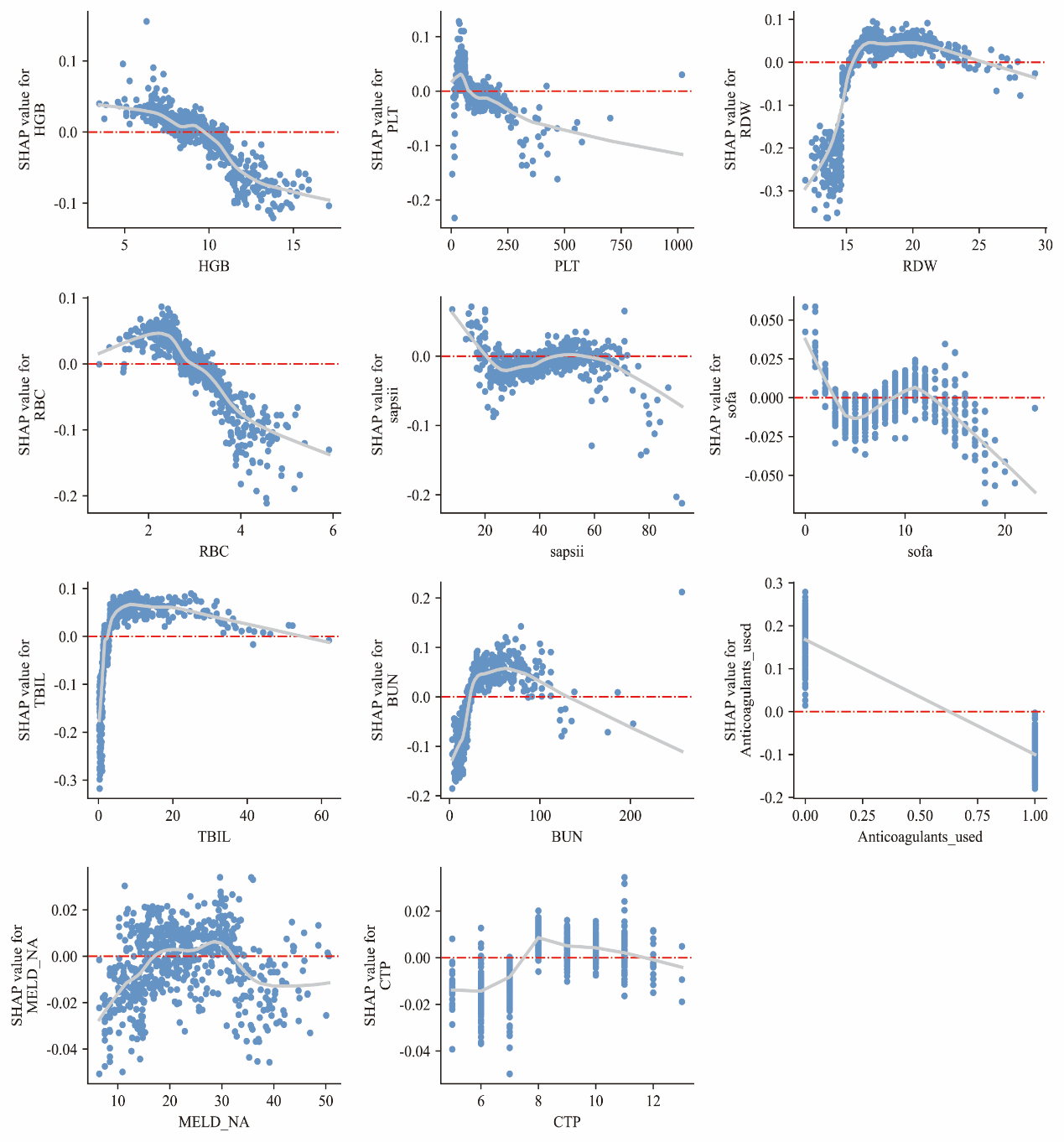


**Fig. S2 Dependence plot of the SHAP variables. The SHAP dependence plot captures the marginal effects of 11 influential variables on model predictions, underscoring the relationship between GIB and its underlying predictors.**


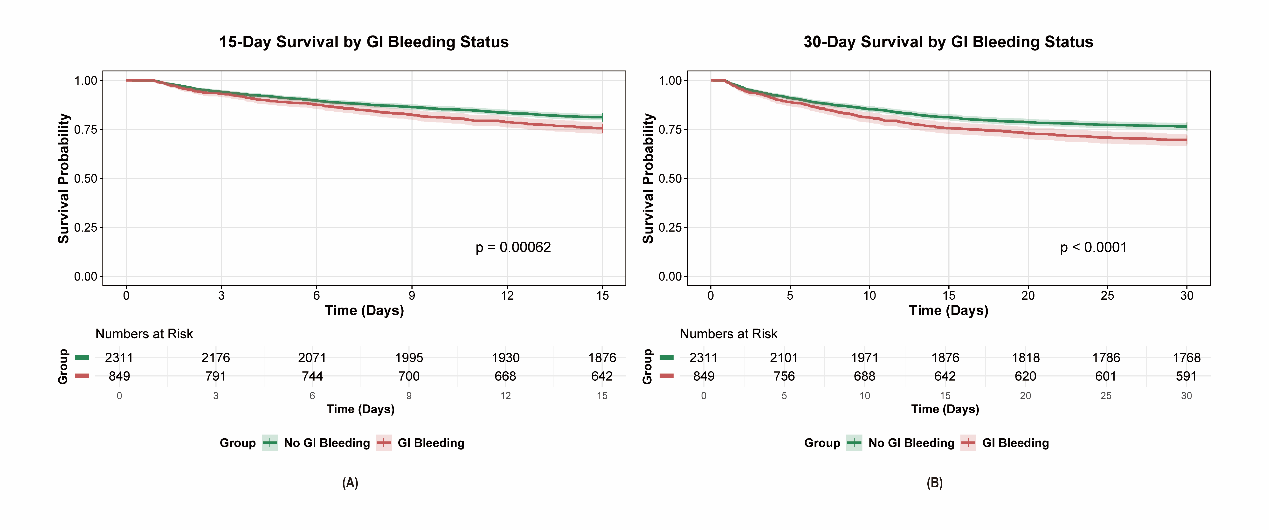


**Fig. S3**. **Kaplan–Meier analysis of short-term survival stratified by the presence of GIB. (A) Survival at 15 days (p = 0.00062). (B) Survival at 30 days (p < 0.0001).**


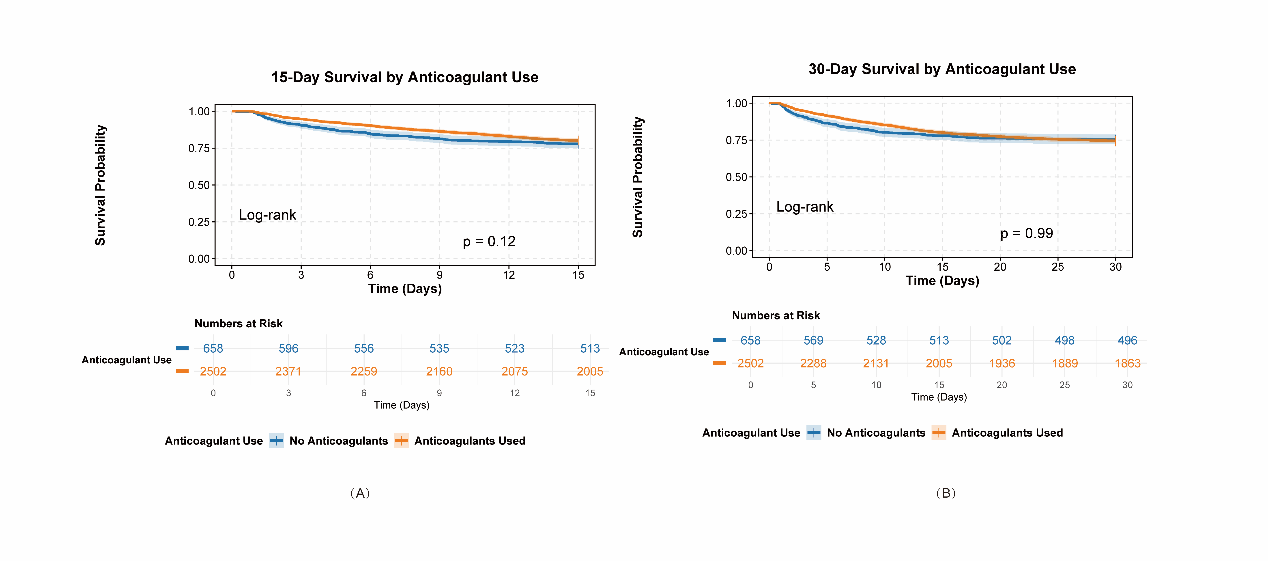


**Fig. S4 Kaplan–Meier analysis of short-term survival stratified by anticoagulant use in all patients with cirrhosis. (A) Survival at 15 days (p = 0.12). (B) Survival at 30 days (p = 0.99). No significant difference in mortality was observed between the two groups.**

**
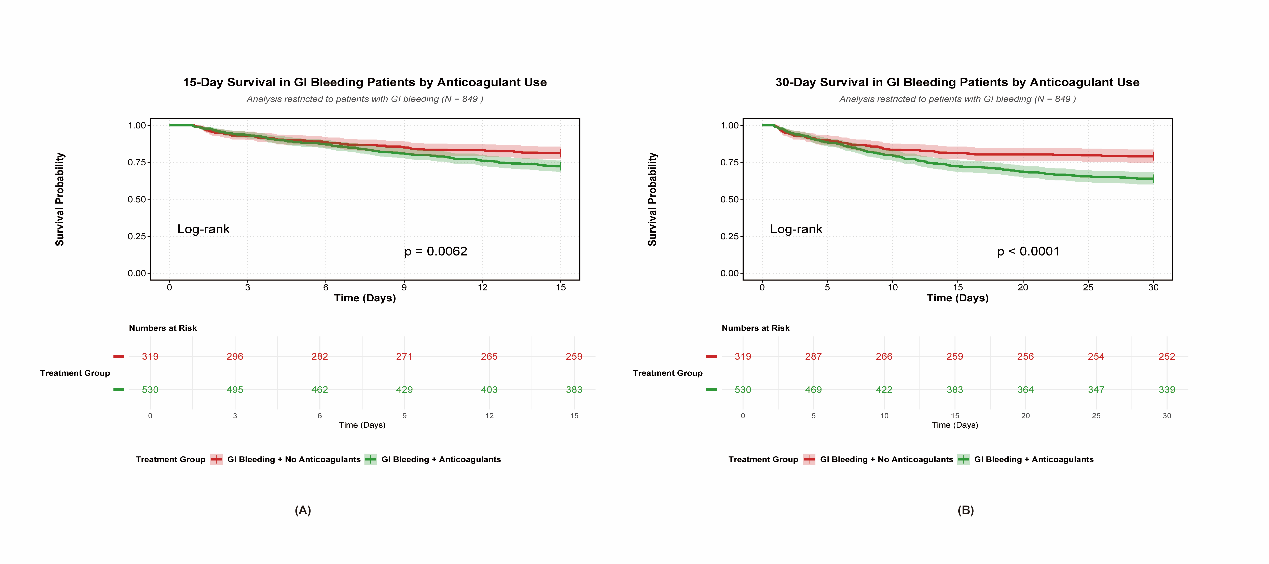
**

**Fig. S5 Kaplan–Meier survival curves for cirrhotic patients with GIB according to anticoagulant therapy. (A) Survival at 15 days (p = 0.0062). (B) Survival at 30 days (p < 0.001).**
